# Genome-wide identification of nitrate-responsive microRNAs by small RNA sequencing in the rice restorer cultivar Nanhui 511

**DOI:** 10.3389/fpls.2023.1198809

**Published:** 2023-06-02

**Authors:** Xiaojian Qin, Xiaowei Li, Cuiping Li, Yuntong Li, Qian Wu, Huan Wen, Dan Jiang, Tingting Tang, Wenbin Nan, Yongshu Liang, Hanma Zhang

**Affiliations:** ^1^ College of Life Sciences, Chongqing Normal University, Chongqing, China; ^2^ Key Laboratory of Molecular Biology of Plants Environmental Adaptations, Chongqing Normal University, Chongqing, China

**Keywords:** *Oryza sativa*, restorer cultivar, nitrogen use efficiency, transcriptome, miRNAs

## Abstract

Rice productivity relies heavily on nitrogen fertilization, and improving nitrogen use efficiency (NUE) is important for hybrid rice breeding. Reducing nitrogen inputs is the key to achieving sustainable rice production and reducing environmental problems. Here, we analyzed the genome-wide transcriptomic changes in microRNAs (miRNAs) in the *indica* rice restorer cultivar Nanhui 511 (NH511) under high (HN) and low nitrogen (LN) conditions. The results showed that NH511 is sensitive to nitrogen supplies and HN conditions promoted the growth its lateral roots at the seedling stage. Furthermore, we identified 483 known miRNAs and 128 novel miRNAs by small RNA sequencing in response to nitrogen in NH511. We also detected 100 differentially expressed genes (DEGs), including 75 upregulated and 25 downregulated DEGs, under HN conditions. Among these DEGs, 43 miRNAs that exhibited a 2-fold change in their expression were identified in response to HN conditions, including 28 upregulated and 15 downregulated genes. Additionally, some differentially expressed miRNAs were further validated by qPCR analysis, which showed that *miR443*, *miR1861b*, and *miR166k-3p* were upregulated, whereas *miR395v* and *miR444b.1* were downregulated under HN conditions. Moreover, the degradomes of possible target genes for *miR166k-3p* and *miR444b.1* and expression variations were analyzed by qPCR at different time points under HN conditions. Our findings revealed comprehensive expression profiles of miRNAs responsive to HN treatments in an *indica* rice restorer cultivar, which advances our understanding of the regulation of nitrogen signaling mediated by miRNAs and provides novel data for high-NUE hybrid rice cultivation.

## 1 Introduction

Rice (*Oryza sativa* L.) is a major crop worldwide and provides food to more than half of the global population. Hybrid breeding is an effective way to deal with the global food crisis, which is predicted to worsen with the increasing global population over the next 20 years (Zhao et al., 2015; Beukert et al., 2017). However, modern breeding strategies have focused on obtaining high yields under high nitrogen application rates, resulting in the development of cultivars with low nitrogen-use efficiency and heavy dependency on intensive nitrogen application (Pan et al., 2019; Liu et al., 2021). Consequently, overutilization of nitrogen has several negative ecological impacts, such as soil acidification (Guo et al., 2010; Yan et al., 2022), increased greenhouse gas emissions (Liu et al., 2016), and increased nitrogen leaching (Liang et al., 2014; Yang et al., 2018). Therefore, improving crop nitrogen use efficiency (NUE) and developing excellent restorer cultivars are crucial factors for hybrid seed production and would be beneficial for the environment as well as for farmers (Dobermann and Cassman, 2005; Zhang, 2007; Ahrens et al., 2010; Hakeem et al., 2011; Xu et al., 2012; Chen et al., 2014; Guo et al., 2017). Moreover, guaranteeing food security and sustainable agricultural development using resource-saving and environmentally friendly methods has become a strategic concern over the past decades (Zhang, 2007; Hakeem et al., 2011; Yu et al., 2022). Importantly, elite restorers are beneficial for the F1 generation in hybrid breeding, and the identification and development of high NUE restorer cultivars play important roles in hybrid seed production, which is directly related to final grain yield and sustainable agricultural development (Zhang, 2007; Gong et al., 2020; Hussain et al., 2022; Ouyang et al., 2022; Wang et al., 2022).

In recent years, several small regulatory RNAs have been uncovered and draw a lot of attention because of their important roles in post-transcriptional or translational gene regulation in animals and plants (Hamilton and Baulcombe, 1999; Carrington and Ambros, 2003; Brodersen et al., 2008; Piriyapongsa and Jordan, 2008; Wang et al., 2016). Moreover, microRNAs (miRNAs) are crucial epigenetic regulators of the expression of protein-coding target genes *via* post-transcriptional gene silencing (Bartel, 2004; Allen et al., 2005; Mallory et al., 2005; Jones-Rhoades et al., 2006; Budak and Akpinar, 2015). In plants, the functional validation of miRNAs showed their indispensability in response to various environmental stress influencing plant growth and development, such as drought (Covarrubias and Reyes, 2010; Ferdous et al., 2015; Islam et al., 2022a), heavy metal stress (Aalami et al., 2022; Nguyen and Kim, 2022), high temperature (Li M. Y. et al., 2014; Pan et al., 2017; Wang et al., 2018; Campos et al., 2023), cold (Zhang et al., 2014), and salinity in rice (Covarrubias and Reyes, 2010; Chiang et al., 2016). Furthermore, several miRNAs have been identified and characterized in plants, including *miR167* and *miR174* involved in signaling during root development (Meng et al., 2011; Liang et al., 2015). More importantly, *miR156*, *miR159*, *miR160*, *miR166*, *miR169*, *miR171*, *miR172*, *miR319*, *miR396*, *miR397*, *miR398*, *miR399*, and *miR408*, which are involved in plant growth and development, have been shown to regulate N absorption and assimilation (Xu et al., 2011; Liang et al., 2012; Nguyen et al., 2015; Li et al., 2016; Tiwari et al., 2020; Lin et al., 2022; Lu et al., 2022). Previous studies have summarized the miRNAs involved in plant sensory functions, nutrient uptake, long-distance root transport, and physiological functions related to nutrients (Chiou, 2007; Islam et al., 2022b). However, these studies were mostly limited to transgenic lines with key genes identified in previous studies and focused on ordinary rice materials, while they did not provide a description of the transcriptome-wide miRNA responses to nitrogen availability in rice restorer cultivars. Based on screening a large number of rice cultivars under hydroponic condition supplied with high nitrogen and low nitrogen respectively, our previous study showed that Nanhui511 (NH511) is an excellent sensitive restorer line responding to nitrogen and also exhibits remarkable phenotype and high NUE under nitrogen supplies by measuring N-related enzymes and metabolites (Xiao et al., 2016). In the present study, we investigated the morphological changes in an *indica* rice restorer cultivar NH511 under different nitrogen supply conditions (namely high nitrogen (HN) and low nitrogen (LN) conditions), and we combined small RNA-seq analyses to assess the genome-wide miRNA variations in response to these conditions. Our combined morphological and transcriptomic analyses of the rice restorer cultivar revealed its nitrogen-responsive characteristics. Our findings improved our understanding of the miRNAs involved in the nitrogen response, and provided new insights into high NUE breeding using elite rice restorers.

## 2 Materials and methods

### 2.1 Rice materials and growth conditions

Seeds of the rice cultivar (*Oryza sativa* cv. Nanhui 511 (NH511)) were germinated in Murashige and Skoog (MS) media for 3 d and then transferred to tap water for 3 d before being transferred into a hydroponic solution containing KNO_3_ as the only N source. The seedlings were then divided into two groups and transferred to HN or LN conditions for six days of cultivation. The seedlings were cultivated in a growth chamber under a photoperiod of 12 h/12 h (light/dark) (~230 μmol m^-2^ s^-1^) at 28°C/25°C, and the solution was renewed every 3 d. For the different nitrogen treatment assays and RNA-seq, the seedlings under normal nitrogen (1 mM KNO_3_) were transferred to LN (0.2 mM KNO_3_) and HN solutions (5 mM KNO_3_) for 3 hours, respectively. Seedlings from different N treatments were collected and frozen in liquid nitrogen for RNA extraction. For time-course expression analysis under HN treatment, the seedlings were transferred to HN solutions (5 mM KNO_3_) and RNA samples were collected at different time points under high nitrogen conditions (5 mM nitrate) by TRIzol reagent (Invitrogen, America) according to standard procedures.

### 2.2 RNA isolation and small RNA-seq analysis

Total small RNA was isolated from the samples treated for 3 h and grown under HN and LN conditions using the *mir*Vana™ RNA Isolation Kits (Thermo Fisher, Vilnius, Lithuania), with three biological replications used for this assay. Subsequently, the RNA samples from these replicates were delivered to Biomarker Technologies (Beijing, China) for library construction and small RNA sequencing, after which transcriptome data were obtained and analyzed. The data content has been submitted to GEO database on 2023.4.18, and been publicly released on 2023.4.21. To review GEO accession GSE230023, the following links allow you to view the uploaded data: https://www.ncbi.nlm.nih.gov/geo/query/acc.cgi?acc=GSE230023.

### 2.3 Quantitative RT-PCR

A total of 2 μg of RNA was used for reverse transcription according to standard procedures. The Vazyme miRNA 1st Strand cDNA Synthesis Kit (by stem-loop) (Vazyme, Nanjing, China) was used to synthesize miRNA cDNA. qPCR analysis and experimental data were obtained using a Roche Light Cycler 480 (Roche, Switzerland), after which the expression variation in the N-related genes was calculated. The primers used in this study are listed in **Supplementary Table S5**.

### 2.4 Identification and characterization of differentially expressed genes

Differential expression analyses of the two conditions were performed using the DESeq R package (version 1.10.1) (Love et al., 2014). DESeq provides statistical routines for determining differential expression in digital miRNA expression data, using a model based on a negative binomial distribution. The resulting P values were adjusted using Benjamini and Hochberg’s approach to control for the false discovery rate. miRNAs with an adjusted p<0.05 found by DESeq were assigned as differentially expressed.

### 2.5 miRNA identification and targets prediction

Known and novel miRNAs were identified using the miRDeep2 software (Friedlander et al., 2012), which is a comprehensive software package for miRNA identification that matches reads to the genome based on the distribution information of reads on the precursor sequence. Target genes of the total miRNAs were predicted using the TargetFinder software (Allen et al., 2005). Possible targets of *miR166k-3p* and *miR444b.1* were predicted using psRNATarget (https://www.zhaolab.org/psRNATarget/) under the condition of an expectation value of less than 3.5, respectively.

### 2.6 Gene ontology enrichment, miRNA structure prediction and targets degradome analysis

Gene Ontology (GO) enrichment analysis of the differentially expressed genes (DEGs) was performed using the GOseq R package based on the Wallenius non-central hyper-geometric distribution. The structure of miRNAs were predicted by RNAfold WebServer (http://rna.tbi.univie.ac.at//cgi-bin/RNAWebSuite/RNAfold.cgi) and the targets degradome data were collected and analyzed from TarDB (http://www.biosequencing.cn/TarDB/).

## 3 Results

### 3.1 Morphological variations and small RNA sequencing of the rice restorer cultivar NH511 under different nitrogen supplies

**Figure 1:**
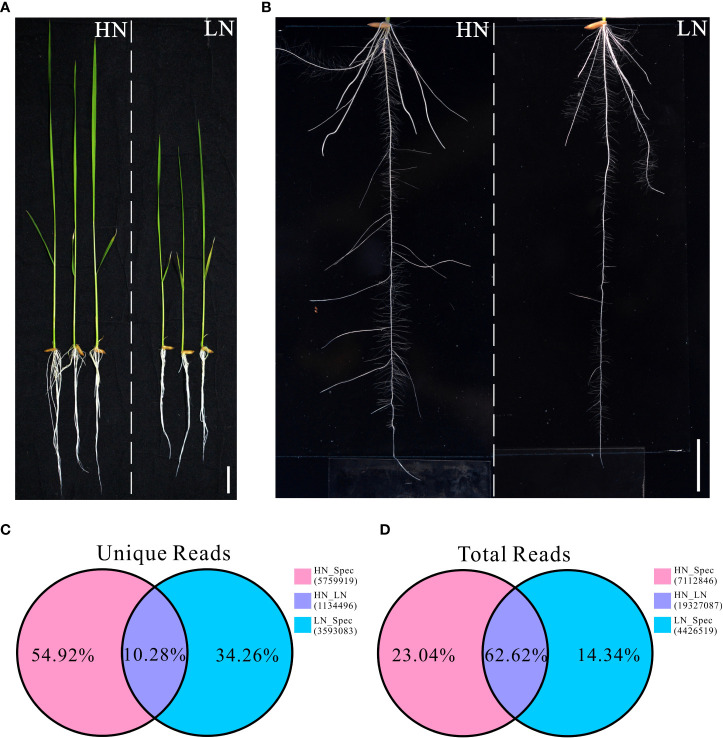
Morphological variations and small RNA sequencing data of Nanhui511 under HN and LN conditions. **(A)** Phenotype of NH511seedlings under HN and LN conditions; Scale bar: 2cm. **(B)** Performance of NH511roots under HN and LN conditions; Scale bar: 1.5cm. **(C)** The proportion of unique reads in different nitrogen conditions; **(D)** The proportion of total reads in different nitrogen conditions.

Our previous study showed that rice cultivars exhibit different morphological and physiological variations when grown under different nitrogen conditions. In the present study, a typical *indica* rice restorer cultivar, namely NH511, was selected for further analysis. To fully understand its different growth responses to different nitrogen supplies, the plants were first germinated and cultivated under normal nitrogen conditions for five days. The seedlings were then divided into two groups and transferred to HN or LN conditions for six days of cultivation. We found that NH511 was sensitive to HN supply conditions, exhibiting promoted growth and increased lateral root numbers and lengths under HN conditions than under LN conditions (**Figures 1A, B**). Moreover, the lateral root tips of NH511 grown under HN and LN conditions were stained with KI-I_2_ and observed under an optical microscope, and our observations further suggested that lateral root development was impaired by influencing root tip maturation under LN conditions (**Figures S1A, B**). Subsequently, transcriptomic read data were obtained from seedlings under HN and LN conditions by small RNA sequencing (**Table S1**), and the statistical data of total reads and unique reads were analyzed. The results showed that the mutually unique reads accounted for 10.28% of unique reads under HN and LN conditions, 54.92% under HN, and 34.26% under LN (**Figure 1C**). For the total reads, the mutual reads accounted for 62.62% of total reads under HN and LN conditions, 23.04% under HN, and 14.34% under LN (**Figure 1D**). Taken together, these transcriptomic data demonstrated that NH511 exhibits favorable variations in miRNA profiles in response to different nitrogen supplies.

### 3.2 Transcriptomic identification and characterization of NH511 miRNA profile in response to different nitrogen supplies

**Figure 2:**
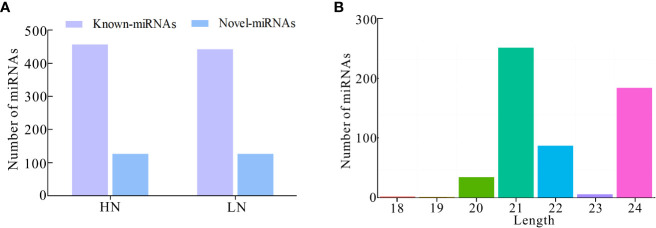
Identification and characterization of miRNAs based on RNA-seq. **(A)** Number of known-miRNAs and novel-miRNAs under HN and LN conditions respectively; **(B)** Number of miRNAs distributed within 18–24 nt in transcriptomic total miRNAs.

Furthermore, we investigated the miRNA profile and characteristics of NH511 in response to different nitrogen conditions. First, the RNA samples extracted from seedlings grown under different nitrogen conditions were sequenced, and the raw sequencing data were processed to obtain unique reads. Furthermore, the filtered data were further processed to facilitate miRNA alignment, identification, and prediction based on the rice genome. A total of 586 miRNAs were identified under HN conditions, including 458 known and 128 novel miRNAs, whereas 572 miRNAs were identified under LN conditions, including 444 known and 128 novel miRNAs (**Figure 2A**). Statistical analysis of the length distribution of clean data was performed based on the analysis and statistics of the original sequencing data. The results showed that in response to nitrogen, most of the miRNAs were distributed within 20–24 nt, with miRNAs with the length of 21 nt and 24 nt occupying a large proportion, which is consistent with the typical length range of miRNAs in plants (**Figure 2B**). Moreover, owing to post-transcriptional base editing of miRNAs, which leads to seed sequence changes and thus changes of target genes, the sequence variations of miRNA were analyzed using isomiRID software (Data sheet2). The target genes of the total miRNAs were predicted and analyzed using the TargetFinder software (**Table S2**). Finally, the miRNA nucleotide bias at each position and the first nucleotide bias for all miRNAs were analyzed, and the results showed that most miRNAs started with A or U at the first nucleotide; A and U also occupied a large proportion of nucleotides at each position (**Figures S2A, B**).

### 3.3 Differential expression analysis of NH511 miRNAs under different nitrogen supplies

**Figure 3:**
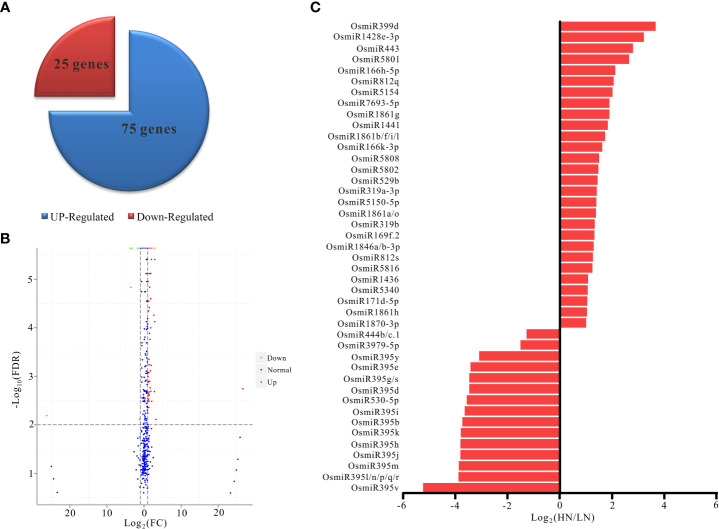
Differentially expressed miRNAs under HN and LN conditions. **(A)** Statistical analysis of miRNAs differentially expressed between HN and LN conditions; **(B)** the Volcano Plot analysis of differentially expressed miRNAs under different nitrogen conditions; **(C)** The over 2-fold differentially expressed miRNAs were identified under HN and LN conditions.

The observed differences in the performance of NH511 under HN and LN conditions prompted us to identify differentially expressed miRNAs under different nitrogen conditions. Therefore, the miRNA differential expression profiles were obtained from the transcriptome data based on DESeq, which provides statistical routines for determining differential expression in digital miRNA expression data using a model based on negative binomial distribution. Our results showed that a total of 100 miRNAs were differentially expressed between HN and LN conditions, including 75 upregulated and 25 downregulated miRNAs (**Figure 3A**). Hierarchical cluster analysis was also performed for the selected differentially expressed miRNAs under HN and LN conditions (**Figure S3**). Moreover, the Volcano Plot also showed differences in miRNA expression levels and showed that the difference in miRNA expression between the HN and LN conditions was statistically significant (**Figure 3B**). Additionally, small RNA sequencing was used to accurately quantify miRNA abundance in the rice restorer cultivar NH511, and the differentially expressed miRNAs with a relative change greater than 2-fold were identified and defined as N-responsive miRNAs (**Figures 3C** and **S4**).

### 3.4 Gene ontology enrichment and Kyoto Encyclopedia of Genes and Genomes annotation of target genes

**Figure 4:**
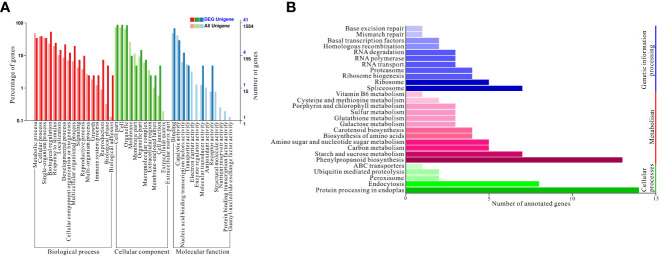
Gene ontology (GO) enrichment and Kyoto Encyclopedia of Genes and Genomes (KEGG) annotations. **(A)** GO enrichment of targets genes of differentially expressed miRNAs; **(B)** KEGG annotation of targets genes of differentially expressed miRNAs under HN and LN conditions.

Based on the above results, a gene ontology (GO) enrichment analysis was performed to further characterize the main biological functions of target genes of differentially expressed miRNAs under high nitrogen supply. All differentially expressed miRNAs were divided into three categories: biological processes, cellular components, and molecular functions. These three categories were further divided into 42 enriched subcategories (**Figure 4A**). To fully understand the cellular pathways of target genes differentially expressed miRNAs under HN and LN conditions, an enrichment analysis based on KEGG was performed. Three KEGG pathway categories were identified: cellular processes, metabolism, and genetic information processing (**Figure 4B**). Specifically, ‘protein processing in endoplasmatic reticulum’ was the most enriched in cellular processes, which could be a consequence of different nitrogen supplies. Regarding metabolism, ‘phenylpropanoid biosynthesis’ was the most overrepresented because phenylpropanoids include a variety of organic compounds synthesized from amino acids such as phenylalanine and tyrosine, which are related to nitrogen metabolism. For genetic information processing, spliceosome, ribosome, and ribosome biogenesis are the top three pathways, which probably influences the gene expression and protein coding involved in nitrogen uptake and assimilation.

### 3.5 Quantitative RT-PCR validation for differentially expressed miRNAs

**Figure 5:**
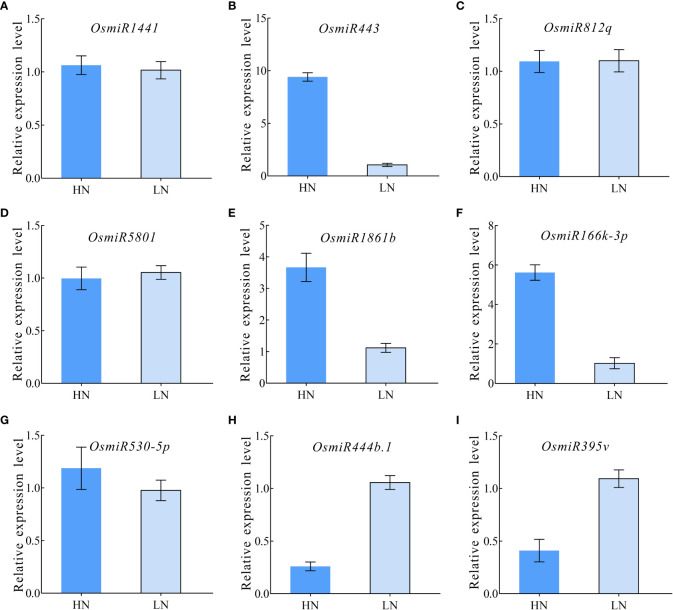
qRT-PCR validation for differentially expressed miRNAs. **(A)** *OsmiRNA1441*; **(B)** *OsmiRNA443*; **(C)** *OsmiRNA812q*; **(D)** *OsmiRNA5801*; **(E)** *OsmiRNA1861b*; **(F)** *OsmiRNA166k-3p*; **(G)** *OsmiRNA530-5p*; **(H)** *OsmiRNA444b.1*; **(I)** *OsmiRNA395v*.

To further validate the differential miRNA expression under HN and LN conditions, a qPCR expression analysis was performed, and the nine selected typical miRNAs (*miR1441*, *miR443*, *miR812q*, *miR5801*, *miR1861b*, *miR166k-3p*, *miR530-5p*, *miR444b.1*, and *miR395v*) were validated using qPCR primers under HN and LN conditions (**Table S5**). The results showed that most miRNAs had an expression pattern similar to that of their small RNA sequencing profiles (**Figures 5A–I**). Furthermore, three miRNAs (*miR443*, *miR1861b*, and *miR166k-3p*) exhibited a significant upregulation pattern in response to HN treatment, but were downregulated expressed in the LN treatment (**Figures 5B, E, F**). The expression of *miR444b.1* and *miR395v* was downregulated under HN conditions but upregulated under LN conditions (**Figure 5H, I**). These data provide a foundation for the functional elucidation of candidate miRNAs and the possibility of selecting elite rice restorers for high-NUE rice breeding.

### 3.6 Target prediction and degradome analysis of *Osa-miR166k-3p* and *Osa-miR444b.1*

**Figure 6:**
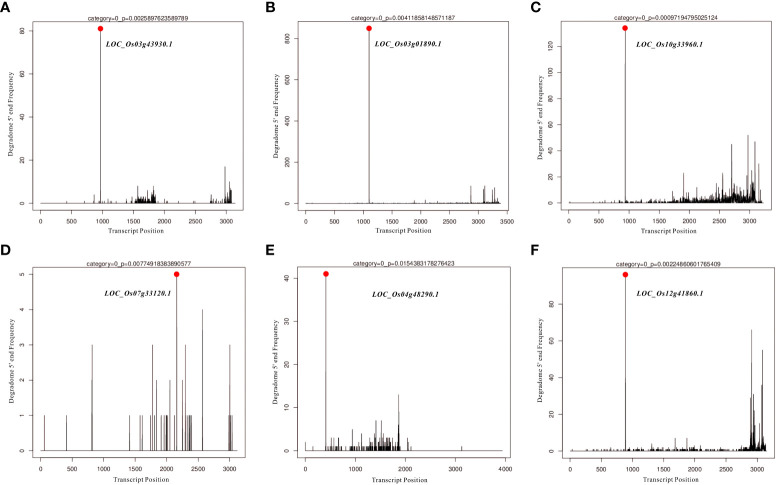
The degradome analysis of target genes for *OsamiR166k-3p*. **(A)** *Os03g43930*; **(B)** *Os03g01890*; **(C)** *Os10g33960*; **(D)** *Os07g33120*; **(E)** *Os04g48290*; **(F)** *Os12g41860*.

**Figure 7:**
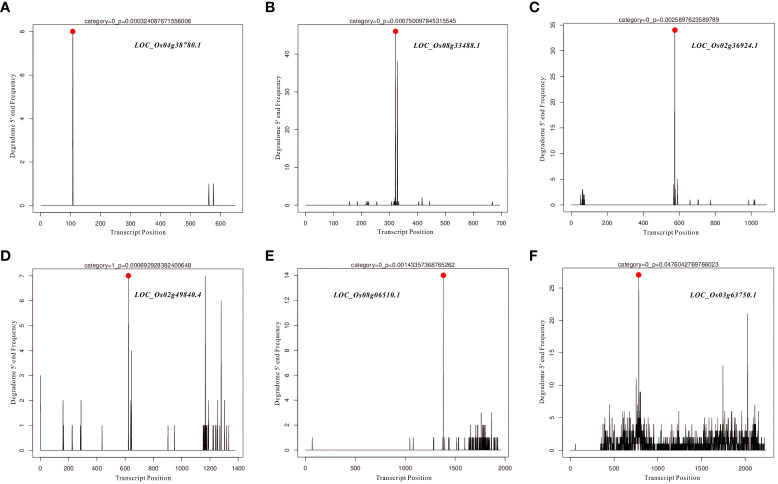
The degradome analysis of target genes for *OsamiR444b.1*. **(A)** *Os04g38780*; **(B)** *Os08g33488*; **(C)** *Os02g36924*; **(D)** *Os02g49840*; **(E)** *Os08g06510*; **(F)** *Os03g63750*.

In the present study, differentially expressed miRNAs were identified from transcriptomic data under HN and LN conditions, and partially representative miRNAs were validated by qRT-PCR analysis. Based on qRT-PCR results and previous studies on identification and characterization of targets genes of these validated miRNAs, we selected *miR166k-3p* and *miR444b.1* for the further investigation. The two candidates, *miR166k-3p* and *miR444b.1*, were characterized and shown to be upregulated and downregulated under HN conditions, respectively. First, possible targets of *miR166k-3p* and *miR444b.1* were predicted using psRNATarget (https://www.zhaolab.org/psRNATarget/) under the condition of an expectation value of less than 3.5, respectively (**Tables S3** and **S4**). As shown by the results, *miR166k-3p* mainly regulated homeodomain containing proteins and other unknown expressed proteins. Homeodomain containing proteins are a large family of transcription factors (TFs) that contain a highly conserved DNA-binding domain of 60 amino acids known as the homeodomain. They mediate protein-protein or protein-DNA interactions during plant growth and development. Furthermore *miR444b.1* regulated MADS-box family proteins, which have been reported to be involved in many important pathways of environmental adaptation in plants. Furthermore, the secondary structures of *miR166k-3p* and *miR444b.1* were predicted and analyzed for the first time by RNAfold WebServer (http://rna.tbi.univie.ac.at//cgi-bin/RNAWebSuite/RNAfold.cgi) (**Figure S5**), and the degradomes of crucial targets were predicted and identified suing the TarDB database (http://www.biosequencing.cn/TarDB/) for *miR166k-3p* (**Figure 6**) and *miR444b.1* (**Figure 7**) respectively. These results provided detailed information on the target gene processing by the two candidate miRNAs as well as a foundation for further studies.

### 3.7 Time-course expression analysis of *Osa-miR166k-3p* and *Osa-miR444b.1* under high nitrogen conditions

**Figure 8:**
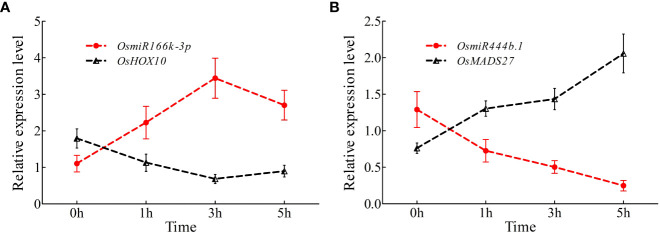
Time-course expression analysis of *OsamiR166k-3p* and *OsamiR444b.1* under high nitrogen conditions. **(A)** Expression analysis of *OsamiR166k-3p* and its target gene *OsHOX10*; **(B)** Expression analysis of *OsamiR444b.1* and its target gene *OsMADS27*.

To further investigate the expression variations in *miR166k-3p* and *miR444b.1* under high nitrogen supply, a qRT-PCR assay was carried out. The expression levels of *miR166k-3p* with its target gene *OsHOX10* (LOC_03g01890) and *miR444b.1* (LOC_02g36924) with its target gene *OsMADS27* under the HN treatment were validated using qPCR primers at different time points. The results showed that *miR166k-3p* expression was upregulated in response to HN condition, which was reversed in the expression pattern of the target gene *OsHOX10* (**Figure 8A**). Similarly, the expression of *miR444b.1* was suppressed, and the expression of its target gene *OsMADS27* was induced under the HN treatment (**Figure 8B**). Taken together, these results further elucidated the response of the two candidate miRNAs (*miR166k-3p* and *miR444b.1*) to nitrogen supplies as well as their different expression patterns, which provides possibilities for further studies of these two candidate miRNAs and new insights for high-NUE rice breeding using elite restorer cultivars.

## 4 Discussion

Rice is one of the most important cereal crops and provides food for half of the world’s population (Liang et al., 2014; Beukert et al., 2017; Liu et al., 2021). Hybrid rice has been successfully developed using a two- or three-line system based on a male sterile line, maintainer line, and restorer line, which has greatly contributed to world food production in the past decades (Chen et al., 2022; Hussain et al., 2022; Jiang et al., 2022; Li et al., 2022). Nanhui 511 (NH511), a typical *indica* rice restorer cultivar, was developed by hybridizing two heavy panicle-type restorer lines, namely Shuhui 881 and Shuhui 527. NH511 has been widely used in hybrid seed production as a restorer line because of its good combining ability and grain quality in hybrid rice breeding, and has been used to breed many hybrid rice cultivars in the past (Wang et al., 2019; Ren et al., 2021).

Nitrogen is a crucial nutrient required for plant growth and development, particularly in crops (Hakeem et al., 2011; Sultana et al., 2020). Nitrogen deficiency and low nitrogen utilization impair crop growth and development, which influences crop yield and food security (Ahrens et al., 2010; Xu et al., 2012; Kabange et al., 2021; Liu et al., 2021). In crop production, excess nitrogen is inevitably leached into water systems and lost to the atmosphere, resulting in severe agricultural environmental problems (Zhang, 2007; Ahrens et al., 2010; Cai et al., 2012). Reducing the amounts of nitrogen fertilizers used in agriculture and cultivating cultivars with high NUE are efficient ways of solving environmental problems and ensuring sustainable crop production. In the present study, NH511 was selected to identify its miRNAs in response to nitrogen. First, morphological variations and nitrogen-responsive miRNAs were first identified and investigated under HN and LN conditions. The results showed that NH511 is a nitrogen-responsive restorer line whose seedling growth was promoted by the regulation of lateral root development and plant height (**Figures 1A, B**). Subsequently, the RNA samples extracted from seedlings grown under different nitrogen conditions were sequenced, and the filtered data were processed to facilitate miRNA alignment, identification, and prediction based on the rice genome. Additionally, miRNA differential expression profiles were obtained from the transcriptome data based on DESeq (**Figures 3A–C**), and most miRNAs showed a similar expression pattern to that of their small RNA sequencing profile according to qPCR assay results (**Figures 5A–I**), implying that we obtained high-quality transcriptomic data and successfully identified the candidate miRNAs in response to different nitrogen supplies. Furthermore, two candidate miRNAs, namely *miR166k-3p* and *miR444b.1*, were chosen for further investigation, and their possible targets were identified and analyzed, and also time-course expression were validated using qPCR primers at different time points under the HN treatment (**Figures 6**, **7**, and **8**). In plants, the *miR166* family comprises multiple members and is a highly conserved family of miRNAs with conserved target genes, the Class III homeodomain-leucine zipper (HD-ZIP III) transcription factors. Some studies showed *miR166* family plays a role in environmental adaptations and plant development, such as drought resistance in rice (Zhang et al., 2018) and diverse developmental pathways (Itoh et al., 2008). Moreover, *miR166* was also reported in adapting to infection by the rice blast fungus *M. oryzae* or to differentially accumulate in blast-resistant and blast-susceptible rice varieties (Li Y. et al., 2014; Salvador et al., 2018). The *miR444* gene family was first discovered in rice and consists of six gene loci (*Osa-MIR444a* to *Osa-MIR444f*), and *miR444* has multiple abundant isoforms and iso-miRs (Jiao et al., 2020). Multiple mature forms of *miR444* are involved in nitrogen stress, and *miR444* gene family might have evolved by sharing stress response elements in both *miR444* isoforms and its targets, such as *miR444-OsMADS27* regulatory module involved in nitrate-dependent root development in rice (Pachamuthu et al., 2022).Taken together, our results further elucidated the response of the two candidate miRNAs, *miR166k-3p* and *miR444b.1*, to different nitrogen supplies, along with a description of their different expression patterns, which provides possibilities for further studies of the two candidate miRNAs involved in nitrogen utilization pathways and high-NUE hybrid rice cultivars cultivation. In summary, this study revealed a comprehensive expression profile of miRNAs responsive to high-nitrogen treatments in the *indica* rice restorer cultivar NH511, which advanced our understanding of nitrogen signaling mediated by miRNAs and provided new insights for high-NUE rice breeding based on elite restorer cultivars.

## 5 Conclusions

We investigated the morphological changes and transcriptomic miRNA variations in the *indica* rice restorer line NH511 under high (HN) and low nitrogen (LN) conditions. Based on our findings, we discussed and compared their nitrogen-responsive characteristics and differentially expressed miRNAs under HN and LN conditions. We further validated the candidate miRNAs and elucidated their response to different nitrogen conditions. We believe that our study makes a significant contribution to rice breeding because we described and identified the N-responsive characteristics and differentially expressed miRNAs in an important rice restorer cultivar under different nitrogen supplies, thus providing novel data for the breeding of high-NUE rice cultivars in the future.

## Data availability statement

The datasets presented in this study can be found in online repositories. The names of the repository/repositories and accession number(s) can be found below: https://www.ncbi.nlm.nih.gov/geo/, GSE230023.

## Author contributions

XQ and HZ designed the experiment. XQ wrote the paper and HZ revised the manuscript. YL, WN and HW validated the results. XL, QW, YL, CL, DJ, and TT contributed to experiments and the acquisition of data. All authors contributed to the article and approved the submitted version.

## References

[B1] Aalami A. H. Hoseinzadeh M. Hosseini Manesh P. Jiryai Sharahi A. Kargar Aliabadi E. (2022). Carcinogenic effects of heavy metals by inducing dysregulation of microRNAs: a review. Mol. Biol. Rep. 49 (12), 12227–12238. doi: 10.1007/s11033-022-07897-x 36269534

[B2] Ahrens T. D. Lobell D. B. Ortiz-Monasterio J. I. Li Y. Matson P. A. (2010). Narrowing the agronomic yield gap with improved nitrogen use efficiency: a modeling approach. Ecol. Appl. 20 (1), 91–100. doi: 10.1890/08-0611.1 20349832

[B3] Allen E. Xie Z. Gustafson A. M. and Carrington J. C. (2005). microRNA-directed phasing during trans-acting siRNA biogenesis in plants. Cell 121 (2), 207–221. doi: 10.1016/j.cell.2005.04.004 15851028

[B4] Bartel D. P. (2004). MicroRNAs: genomics, biogenesis, mechanism, and function. Cell 116 (2), 281–297. doi: 10.1016/s0092-8674(04)00045-5 14744438

[B5] Beukert U. Li Z. Liu G. Zhao Y. Ramachandra N. Mirdita V.. (2017). Genome-based identification of heterotic patterns in rice. Rice (N Y) 10 (1), 22. doi: 10.1186/s12284-017-0163-4 28527137 PMC5438337

[B6] Brodersen P. Sakvarelidze-Achard L. Bruun-Rasmussen M. Dunoyer P. Yamamoto Y. Y. Sieburth L.. (2008). Widespread translational inhibition by plant miRNAs and siRNAs. Science 320 (5880), 1185–1190. doi: 10.1126/science.1159151 18483398

[B7] Budak H. Akpinar B. A. (2015). Plant miRNAs: biogenesis, organization and origins. Funct. Integr. Genomics 15 (5), 523–531. doi: 10.1007/s10142-015-0451-2 26113396

[B8] Cai H. Lu Y. Xie W. Zhu T. Lian X. (2012). Transcriptome response to nitrogen starvation in rice. J. Biosci. 37 (4), 731–747. doi: 10.1007/s12038-012-9242-2 22922198

[B9] Campos C. Coito J. L. Cardoso H. Marques da Silva J. Pereira H. S. Viegas W.. (2023). Dynamic regulation of grapevine’s microRNAs in response to mycorrhizal symbiosis and high temperature. Plants (Basel) 12, (5). doi: 10.3390/plants12050982 PMC10005052 36903843

[B10] Carrington J. C. Ambros V. (2003). Role of microRNAs in plant and animal development. Science 301 (5631), 336–338. doi: 10.1126/science.1085242 12869753

[B11] Chen R. Deng Y. Ding Y. Guo J. Qiu J. Wang B.. (2022). Rice functional genomics: decades’ efforts and roads ahead. Sci. China Life Sci. 65 (1), 33–92. doi: 10.1007/s11427-021-2024-0 34881420

[B12] Chen S. Wang D. Xu C. Ji C. Zhang X. Zhao X.. (2014). Responses of super rice (Oryza sativa l.) to different planting methods for grain yield and nitrogen-use efficiency in the single cropping season. PloS One 9 (8), e104950. doi: 10.1371/journal.pone.0104950 25111805 PMC4128727

[B13] Chiang C. P. Yim W. C. Sun Y. H. Ohnishi M. Mimura T. Cushman J. C.. (2016). Identification of ice plant (Mesembryanthemum crystallinum l.) MicroRNAs using RNA-seq and their putative roles in high salinity responses in seedlings. Front. Plant Sci. 7. doi: 10.3389/fpls.2016.01143 PMC4977306 27555850

[B14] Chiou T. J. (2007). The role of microRNAs in sensing nutrient stress. Plant Cell Environ. 30 (3), 323–332. doi: 10.1111/j.1365-3040.2007.01643.x 17263777

[B15] Covarrubias A. A. Reyes J. L. (2010). Post-transcriptional gene regulation of salinity and drought responses by plant microRNAs. Plant Cell Environ. 33 (4), 481–489. doi: 10.1111/j.1365-3040.2009.02048.x 19781008

[B16] Dobermann A. Cassman K. G. (2005). Cereal area and nitrogen use efficiency are drivers of future nitrogen fertilizer consumption. Sci. China C Life Sci. 48 Spec No, 745–758. doi: 10.1007/BF03187115 16512198

[B17] Ferdous J. Hussain S. S. Shi B. J. (2015). Role of microRNAs in plant drought tolerance. Plant Biotechnol. J. 13 (3), 293–305. doi: 10.1111/pbi.12318 25583362 PMC6680329

[B18] Friedlander M. R. Mackowiak S. D. Li N. Chen W. Rajewsky N. (2012). miRDeep2 accurately identifies known and hundreds of novel microRNA genes in seven animal clades. Nucleic Acids Res. 40 (1), 37–52. doi: 10.1093/nar/gkr688 21911355 PMC3245920

[B19] Gong Z. Xiong L. Shi H. Yang S. Herrera-Estrella L. R. Xu G.. (2020). Plant abiotic stress response and nutrient use efficiency. Sci. China Life Sci. 63 (5), 635–674. doi: 10.1007/s11427-020-1683-x 32246404

[B20] Guo J. Hu X. Gao L. Xie K. Ling N. Shen Q.. (2017). The rice production practices of high yield and high nitrogen use efficiency in jiangsu, China. Sci. Rep. 7 (1), 2101. doi: 10.1038/s41598-017-02338-3 28522870 PMC5437039

[B21] Guo J. H. Liu X. J. Zhang Y. Shen J. L. Han W. X. Zhang W. F.. (2010). Significant acidification in major Chinese croplands. Science 327 (5968), 1008–1010. doi: 10.1126/science.1182570 20150447

[B22] Hakeem K. R. Ahmad A. Iqbal M. Gucel S. Ozturk M. (2011). Nitrogen-efficient rice cultivars can reduce nitrate pollution. Environ. Sci. pollut. Res. Int. 18 (7), 1184–1193. doi: 10.1007/s11356-010-0434-8 21359512

[B23] Hamilton A. J. Baulcombe D. C. (1999). A species of small antisense RNA in posttranscriptional gene silencing in plants. Science 286 (5441), 950–952. doi: 10.1126/science.286.5441.950 10542148

[B24] Hussain I. Ali S. Liu W. Awais M. Li J. Liao Y.. (2022). Identification of heterotic groups and patterns based on genotypic and phenotypic characteristics among rice accessions of diverse origins. Front. Genet. 13. doi: 10.3389/fgene.2022.811124 PMC8832281 35154278

[B25] Islam W. Idrees A. Waheed A. Zeng F. (2022a). Plant responses to drought stress: microRNAs in action. Environ. Res. 215 (Pt 2), 114282. doi: 10.1016/j.envres.2022.114282 36122702

[B26] Islam W. Tauqeer A. Waheed A. Zeng F. (2022b). MicroRNA mediated plant responses to nutrient stress. Int. J. Mol. Sci. 23, (5). doi: 10.3390/ijms23052562 PMC8910084 35269700

[B27] Itoh J. Hibara K. Sato Y. Nagato Y. (2008). Developmental role and auxin responsiveness of class III homeodomain leucine zipper gene family members in rice. Plant Physiol. 147, 1960–1975. doi: 10.1104/pp.108.118679 18567825 PMC2492597

[B28] Jiang H. Lu Q. Qiu S. Yu H. Wang Z. Yu Z.. (2022). Fujian cytoplasmic male sterility and the fertility restorer gene OsRf19 provide a promising breeding system for hybrid rice. Proc. Natl. Acad. Sci. U.S.A. 119 (34), e2208759119. doi: 10.1073/pnas.2208759119 35969741 PMC9407659

[B29] Jiao X. Wang H. Yan J. Kong X. Liu Y. Chu J.. (2020). Promotion of BR biosynthesis by miR444 is required for ammonium-triggered inhibition of root growth. Plant Physiol. 182 (3), 1454–1466. doi: 10.1104/pp.19.00190 31871071 PMC7054888

[B30] Jones-Rhoades M. W. Bartel D. P. Bartel B. (2006). MicroRNAS and their regulatory roles in plants. Annu. Rev. Plant Biol. 57, 19–53. doi: 10.1146/annurev.arplant.57.032905.105218 16669754

[B31] Kabange N. R. Park S. Y. Lee J. Y. Shin D. Lee S. M. Kwon Y.. (2021). New insights into the transcriptional regulation of genes involved in the nitrogen use efficiency under potassium chlorate in rice (Oryza sativa l.). Int. J. Mol. Sci. 22. doi: 10.3389/fgene.2020.583785 PMC7926690 33671842

[B32] Li P. Chen Y. H. Lu J. Zhang C. Q. Liu Q. Q. Li Q. F. (2022). Genes and their molecular functions determining seed structure, components, and quality of rice. Rice (N Y) 15 (1), 18. doi: 10.1186/s12284-022-00562-8 35303197 PMC8933604

[B33] Li H. Hu B. Wang W. Zhang Z. Liang Y. Gao X.. (2016). Identification of microRNAs in rice root in response to nitrate and ammonium. J. Genet. Genomics 43 (11), 651–661. doi: 10.1016/j.jgg.2015.12.002 27372185

[B34] Li Y. Lu Y. Shi Y. Wu L. Xu Y. Huang F.. (2014). Multiple rice microRNAs are involved in immunity against the blast fungus magnaporthe oryzae. Plant Physiol. 164, 1077–1092. doi: 10.1104/pp.113.230052 24335508 PMC3912081

[B35] Li M. Y. Wang F. Xu Z. S. Jiang Q. Ma J. Tan G. F.. (2014). High throughput sequencing of two celery varieties small RNAs identifies microRNAs involved in temperature stress response. BMC Genomics 15, 242. doi: 10.1186/1471-2164-15-242 24673837 PMC3986682

[B36] Liang G. Ai Q. Yu D. (2015). Uncovering miRNAs involved in crosstalk between nutrient deficiencies in arabidopsis. Sci. Rep. 5, 11813. doi: 10.1038/srep11813 26134148 PMC4488870

[B37] Liang X. Q. Harter T. Porta L. van Kessel C. Linquist B. A. (2014). Nitrate leaching in californian rice fields: a field- and regional-scale assessment. J. Environ. Qual 43 (3), 881–894. doi: 10.2134/jeq2013.10.0402 25602817

[B38] Liang G. He H. Yu D. (2012). Identification of nitrogen starvation-responsive microRNAs in arabidopsis thaliana. PloS One 7 (11), e48951. doi: 10.1371/journal.pone.0048951 23155433 PMC3498362

[B39] Lin Y. Chu S. Xu X. Han X. Huang H. Tong Z.. (2022). Identification of nitrogen starvation-responsive miRNAs to reveal the miRNA-mediated regulatory network in betula luminifera. Front. Genet. 13. doi: 10.3389/fgene.2022.957505 PMC9428261 36061195

[B40] Liu W. Hussain S. Wu L. Qin Z. Li X. Lu J.. (2016). Greenhouse gas emissions, soil quality, and crop productivity from a mono-rice cultivation system as influenced by fallow season straw management. Environ. Sci. pollut. Res. Int. 23 (1), 315–328. doi: 10.1007/s11356-015-5227-7 26304808

[B41] Liu Y. Wang H. Jiang Z. Wang W. Xu R. Wang Q.. (2021). Genomic basis of geographical adaptation to soil nitrogen in rice. Nature 590 (7847), 600–605. doi: 10.1038/s41586-020-03091-w 33408412

[B42] Love M. I. Huber W. Anders S. (2014). Moderated estimation of fold change and dispersion for RNA-seq data with DESeq2. Genome Biol. 15 (12), 550. doi: 10.1186/s13059-014-0550-8 25516281 PMC4302049

[B43] Lu Y. Zhang J. Han Z. Han Z. Li S. Zhang J.. (2022). Screening of differentially expressed microRNAs and target genes in two potato varieties under nitrogen stress. BMC Plant Biol. 22 (1), 478. doi: 10.1186/s12870-022-03866-5 36207676 PMC9547441

[B44] Mallory A. C. Bartel D. P. Bartel B. (2005). MicroRNA-directed regulation of arabidopsis AUXIN RESPONSE FACTOR17 is essential for proper development and modulates expression of early auxin response genes. Plant Cell 17 (5), 1360–1375. doi: 10.1105/tpc.105.031716 15829600 PMC1091760

[B45] Meng Y. Shao C. Wang H. Chen M. (2011). The regulatory activities of plant microRNAs: a more dynamic perspective. Plant Physiol. 157 (4), 1583–1595. doi: 10.1104/pp.111.187088 22003084 PMC3327222

[B46] Nguyen H. D. Kim M. S. (2022). Exposure to a mixture of heavy metals induces cognitive impairment: genes and microRNAs involved. Toxicology 471, 153164. doi: 10.1016/j.tox.2022.153164 35346790

[B47] Nguyen G. N. Rothstein S. J. Spangenberg G. Kant S. (2015). Role of microRNAs involved in plant response to nitrogen and phosphorous limiting conditions. Front. Plant Sci. 6. doi: 10.3389/fpls.2015.00629 PMC4534779 26322069

[B48] Ouyang Y. Li X. Zhang Q. (2022). Understanding the genetic and molecular constitutions of heterosis for developing hybrid rice. J. Genet. Genomics 49 (5), 385–393. doi: 10.1016/j.jgg.2022.02.022 35276387

[B49] Pachamuthu K. Hari Sundar V. Narjala A. Singh R. R. Das S. Avik Pal H. C. Y.. (2022). Nitrate-dependent regulation of miR444-OsMADS27 signalling cascade controls root development in rice. J. Exp. Bot. 73 (11), 3511–3530. doi: 10.1093/jxb/erac083 35243491

[B50] Pan C. Ye L. Zheng Y. Wang Y. Yang D. Liu X.. (2017). Identification and expression profiling of microRNAs involved in the stigma exsertion under high-temperature stress in tomato. BMC Genomics 18 (1), 843. doi: 10.1186/s12864-017-4238-9 29096602 PMC5668977

[B51] Pan J. Zhao J. Liu Y. Huang N. Tian K. Shah F.. (2019). Optimized nitrogen management enhances lodging resistance of rice and its morpho-anatomical, mechanical, and molecular mechanisms. Sci. Rep. 9 (1), 20274. doi: 10.1038/s41598-019-56620-7 31889083 PMC6937289

[B52] Piriyapongsa J. Jordan I. K. (2008). Dual coding of siRNAs and miRNAs by plant transposable elements. RNA 14 (5), 814–821. doi: 10.1261/rna.916708 18367716 PMC2327354

[B53] Ren Y. Chen D. Li W-j Tao L. Yuan G.-q. Cao Y.. (2021). Genome-wide pedigree analysis of elite rice shuhui 527 reveals key regions for breeding. J. Integr. Agric. 20 (1), 35–45. doi: 10.1016/S2095-3119(20)63256-7

[B54] Salvador G. Hsing Y. San Segundo (2018). The polycistronic miR166k-166h ositively regulates rice immunity *via* post-transcriptional control of EIN2. Front. Plant Sci. 9. doi: 10.3389/fpls.2018.00337 PMC5869255 29616057

[B55] Sultana N. Islam S. Juhasz A. Yang R. She M. Alhabbar Z.. (2020). Transcriptomic study for identification of major nitrogen stress responsive genes in Australian bread wheat cultivars. Front. Genet. 11. doi: 10.3389/fgene.2020.583785 PMC7554635 33193713

[B56] Tiwari J. K. Buckseth T. Zinta R. Saraswati A. Singh R. K. Rawat S.. (2020). Genome-wide identification and characterization of microRNAs by small RNA sequencing for low nitrogen stress in potato. PloS One 15 (5), e0233076. doi: 10.1371/journal.pone.0233076 32428011 PMC7237020

[B57] Wang M. Chen J. Zhou F. Yuan J. Chen L. Wu R.. (2022). The ties of brotherhood between japonica and indica rice for regional adaptation. Sci. China Life Sci. 65 (7), 1369–1379. doi: 10.1007/s11427-021-2019-x 34902099

[B58] Wang Y. Guo S. Wang L. Wang L. He X. Shu S.. (2018). Identification of microRNAs associated with the exogenous spermidine-mediated improvement of high-temperature tolerance in cucumber seedlings (Cucumis sativus l.). BMC Genomics 19 (1), 285. doi: 10.1186/s12864-018-4678-x 29690862 PMC5937831

[B59] Wang F. Johnson N. R. Coruh C. Axtell M. J. (2016). Genome-wide analysis of single non-templated nucleotides in plant endogenous siRNAs and miRNAs. Nucleic Acids Res. 44 (15), 7395–7405. doi: 10.1093/nar/gkw457 27207877 PMC5009732

[B60] Wang C. Tang S. Zhan Q. Hou Q. Zhao Y. Zhao Q.. (2019). Dissecting a heterotic gene through GradedPool-seq mapping informs a rice-improvement strategy. Nat. Commun. 10 (1), 2982. doi: 10.1038/s41467-019-11017-y 31278256 PMC6611799

[B61] Xiao J. Yan H. Yang Y. Liang Y. Nan W. Zhang H.. (2016). Screening and research of different rice (Oryza sativa) varieties based on nitrate absorption and utilization in seedlings. Plant Physiol. J. 52 (12), 1941–1949. doi: 10.13592/j.cnki.ppj.2016.0315

[B62] Xu G. Fan X. Miller A. J. (2012). Plant nitrogen assimilation and use efficiency. Annu. Rev. Plant Biol. 63, 153–182. doi: 10.1146/annurev-arplant-042811-105532 22224450

[B63] Xu Z. Zhong S. Li X. Li W. Rothstein S. J. Zhang S.. (2011). Genome-wide identification of microRNAs in response to low nitrate availability in maize leaves and roots. PloS One 6 (11), e28009. doi: 10.1371/journal.pone.0028009 22132192 PMC3223196

[B64] Yan C. Liu Y. Cui X. Cao L. Xiong J. Zhang Q.. (2022). Corrigendum to “Improving the efficiency of anaerobic digestion: domesticated paddy soil microbes enhances the hydrolytic acidification of rice straw and pig manure” [Bioresour. technol. 345 (2022) 126570]. Bioresour Technol. 348, 126821. doi: 10.1016/j.biortech.2022.126821 35153124

[B65] Yang S. Wang Y. Liu R. Xing L. Yang Z. (2018). Improved crop yield and reduced nitrate nitrogen leaching with straw return in a rice-wheat rotation of ningxia irrigation district. Sci. Rep. 8 (1), 9458. doi: 10.1038/s41598-018-27776-5 29930282 PMC6013432

[B66] Yu S. Ali J. Zhou S. Ren G. Xie H. Xu J.. (2022). From green super rice to green agriculture: reaping the promise of functional genomics research. Mol. Plant 15 (1), 9–26. doi: 10.1016/j.molp.2021.12.001 34883279

[B67] Zhang Q. (2007). Strategies for developing green super rice. Proc. Natl. Acad. Sci. U.S.A. 104 (42), 16402–16409. doi: 10.1073/pnas.0708013104 17923667 PMC2034246

[B68] Zhang J. Zhang H. Srivastava A. Pan Y. Bai J. Fang J.. (2018). Knockdown of rice microRNA166 confers drought resistance by causing leaf rolling and altering stem xylem development. Plant Physiol. 176 (3), 2082–2094. doi: 10.1104/pp.17.01432 29367235 PMC5841683

[B69] Zhang Y. Zhu X. Chen X. Song C. Zou Z. Wang Y.. (2014). Identification and characterization of cold-responsive microRNAs in tea plant (Camellia sinensis) and their targets using high-throughput sequencing and degradome analysis. BMC Plant Biol. 14, 271. doi: 10.1186/s12870-014-0271-x 25330732 PMC4209041

[B70] Zhao Y. Li Z. Liu G. Jiang Y. Maurer H. P. Wurschum T.. (2015). Genome-based establishment of a high-yielding heterotic pattern for hybrid wheat breeding. Proc. Natl. Acad. Sci. U.S.A. 112 (51), 15624–15629. doi: 10.1073/pnas.1514547112 26663911 PMC4697414

