# Supplementary figures and images for: Genome-wide identification of nitrate-responsive microRNAs by small RNA sequencing in the rice restorer cultivar Nanhui 511
Source: Front Plant Sci. 2023 Jun 2;14:1198809. doi: 10.3389/fpls.2023.1198809 (PMC10272429; doi:10.3389/fpls.2023.1198809)

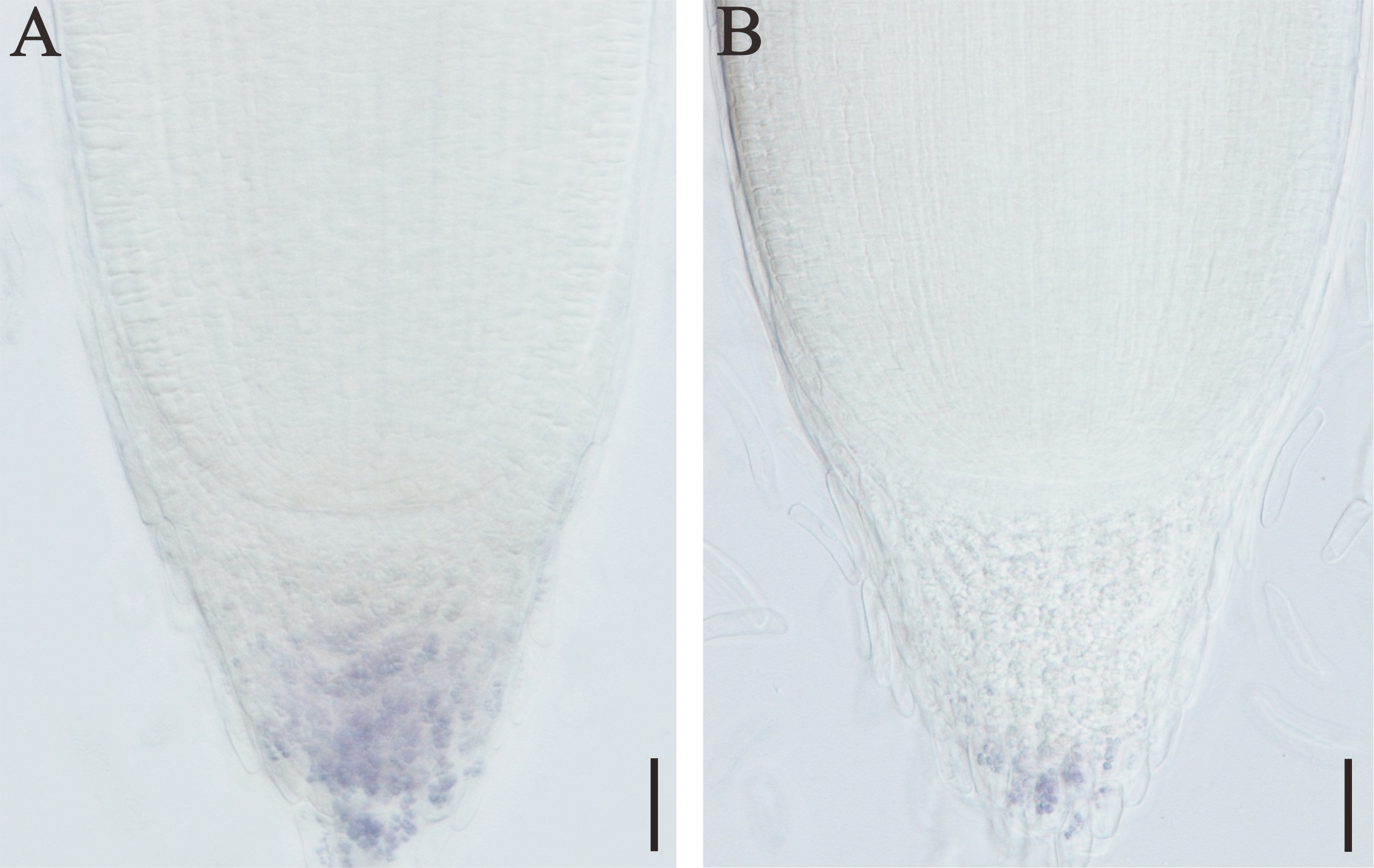

Supplement: Supplementary file 2 [file Image_1.jpg]

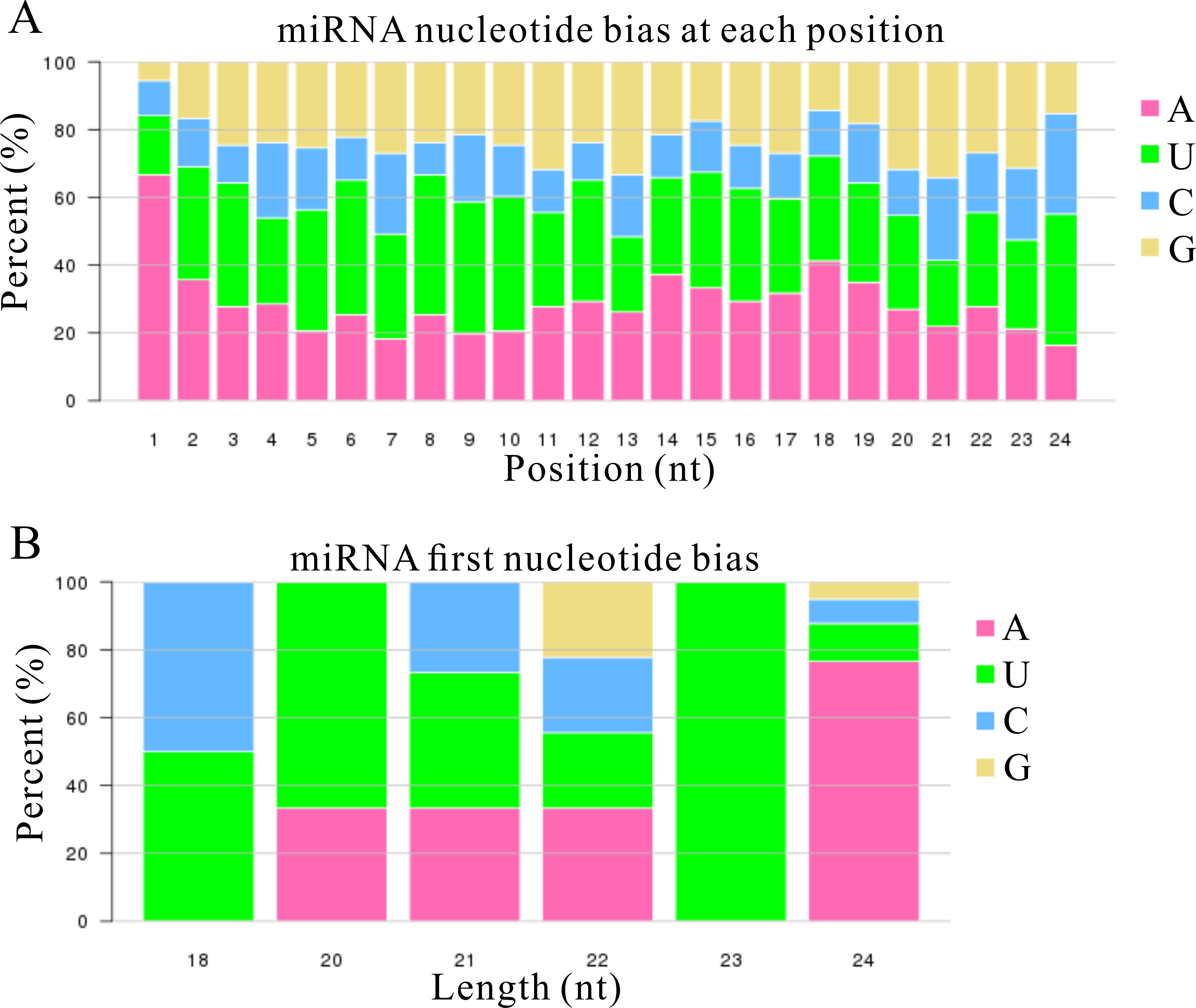

Supplement: Supplementary file 3 [file Image_2.jpg]

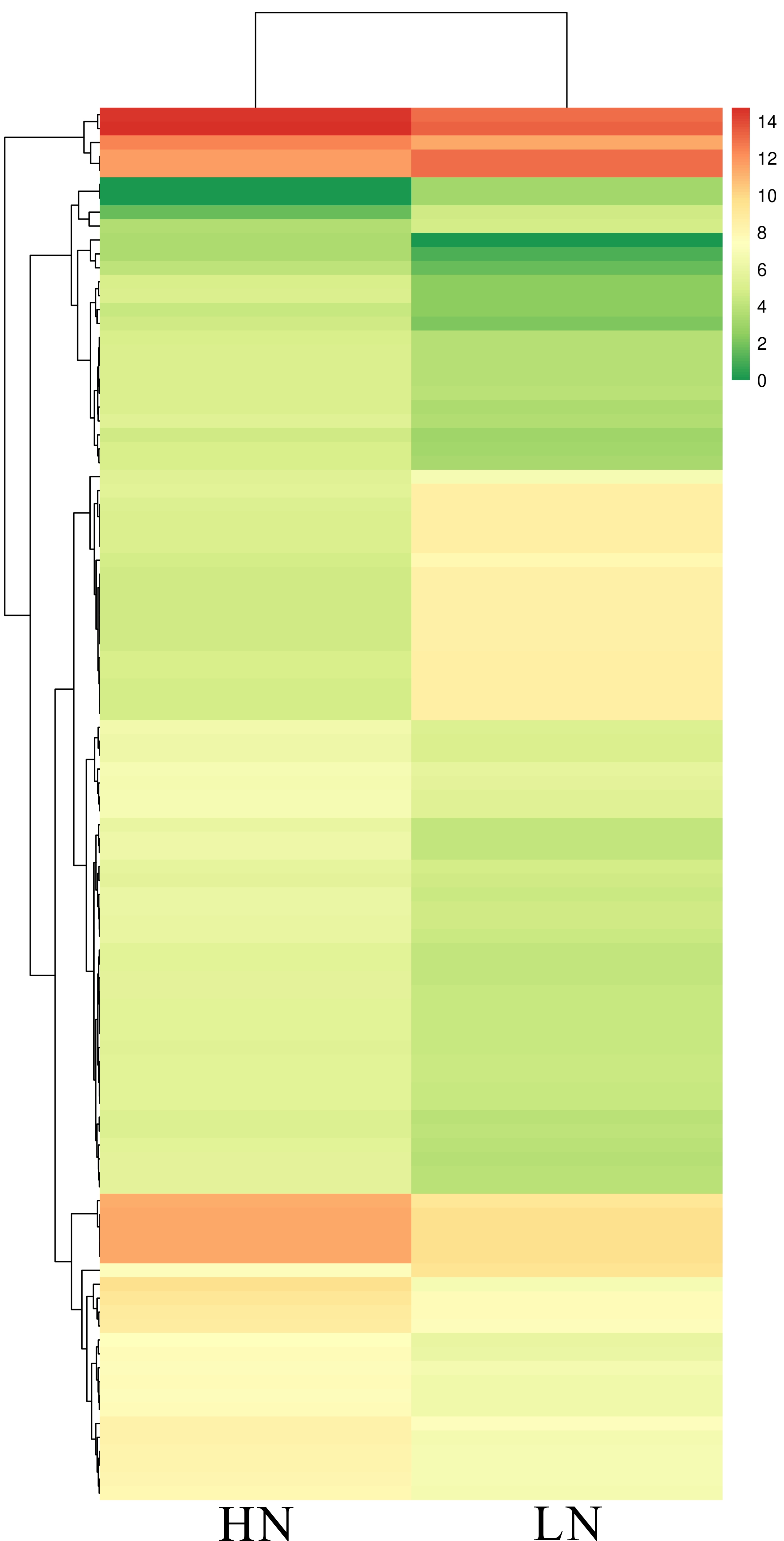

Supplement: Supplementary file 4 [file Image_3.jpg]

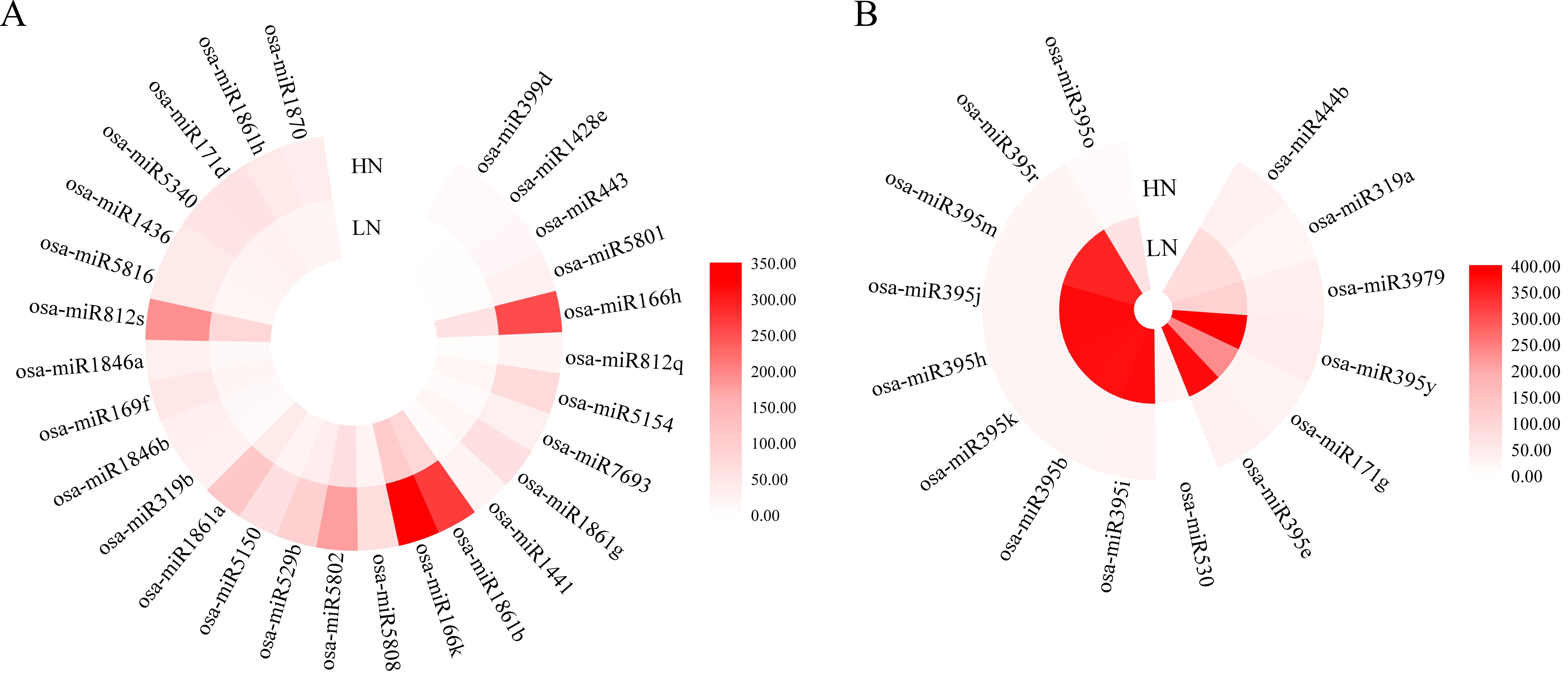

Supplement: Supplementary file 5 [file Image_4.jpg]

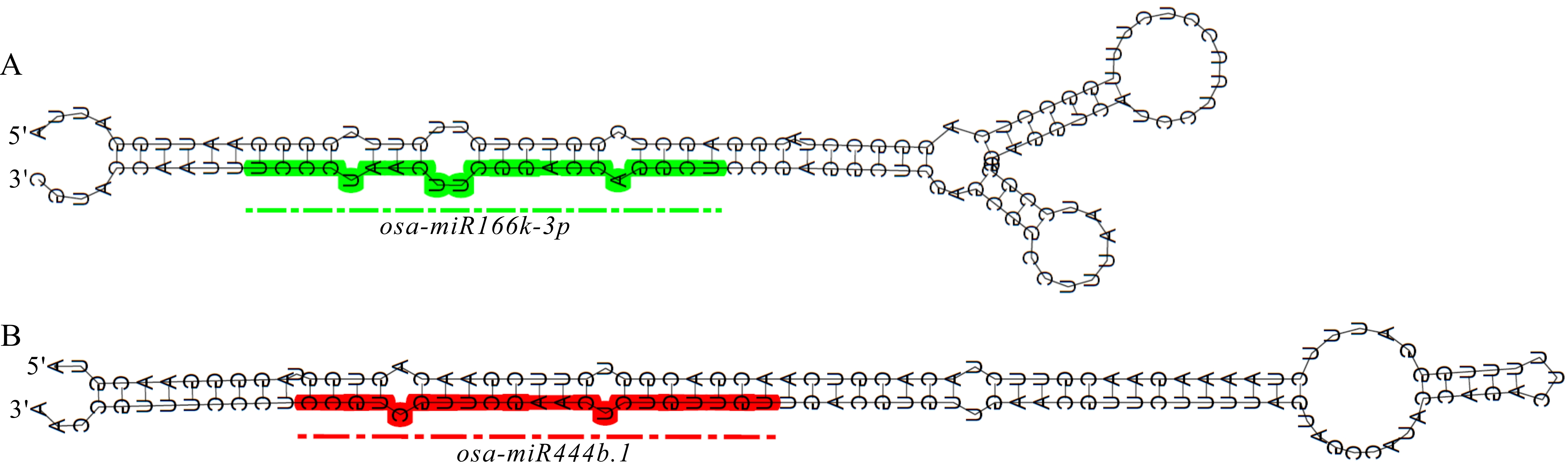

Supplement: Supplementary file 6 [file Image_5.jpg]
