# Supplementary material for: Genome-wide identification of nitrate-responsive microRNAs by small RNA sequencing in the rice restorer cultivar Nanhui 511
Source: Front Plant Sci. 2023 Jun 2;14:1198809. doi: 10.3389/fpls.2023.1198809 (PMC10272429; doi:10.3389/fpls.2023.1198809)
Supplement: Supplementary file 7 [file DataSheet_1.docx]

Supplementary Material

Genome-wide identification of nitrate-responsive microRNAs by small RNA sequencing in the rice restorer cultivar Nanhui 511

Xiaojian Qin^1, 2, *^, Xiaowei Li^1^, Qian Wu^1^, Cuiping Li^1^, Yuntong Li^1^, Dan Jiang^1^, Tingting Tang^1^, Wenbin Nan^1, 2^, Yongshu Liang^1, 2^, Hanma Zhang^1, 2^

*** Correspondence:** Xiaojian Qin Email: [xiaojianqin_ab@163.com](mailto:xiaojianqin_ab@163.com).

# Supplementary Figures and Tables

## Supplementary Figures


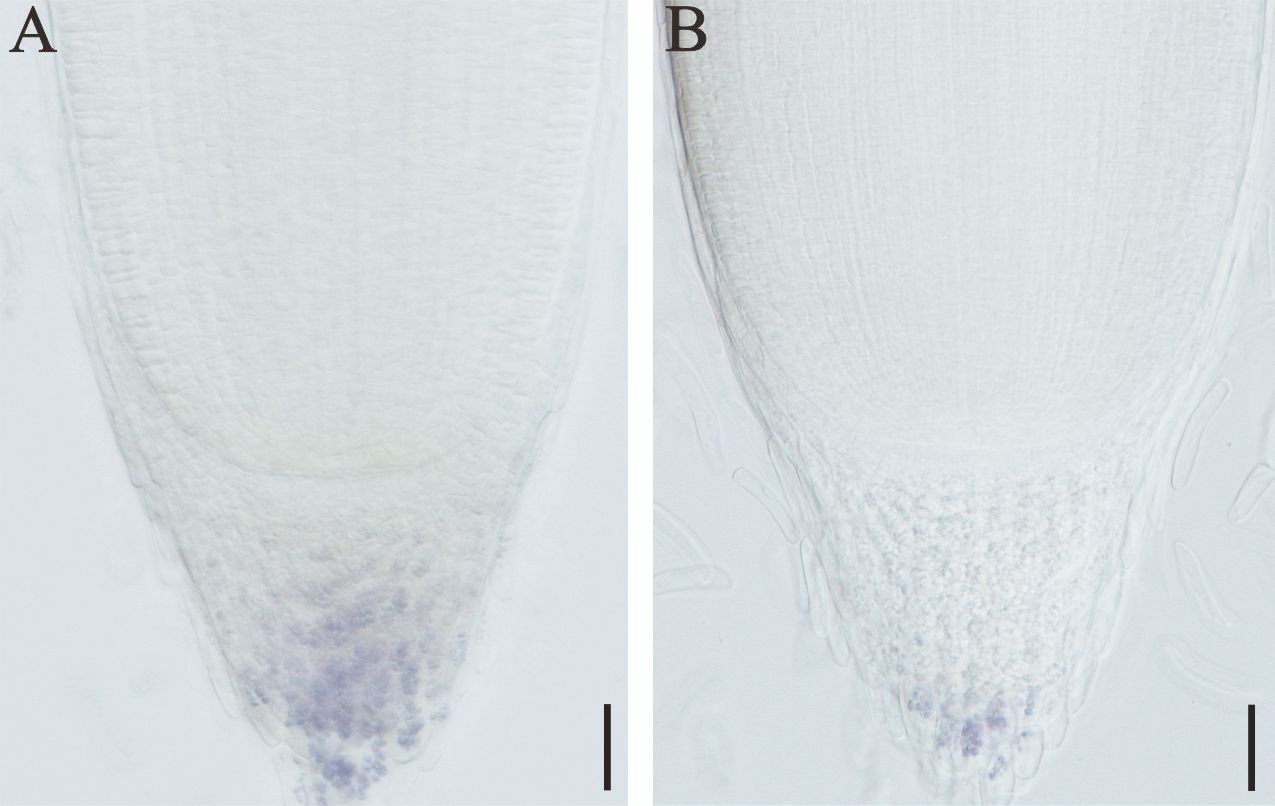


**Supplementary Figure 1.** The microscope observation of lateral root tip of rice cultivar Nanhui511 under HN and LN conditions. **(A)** HN condition; **(B)** LN condition. Scale bar: 20μm.


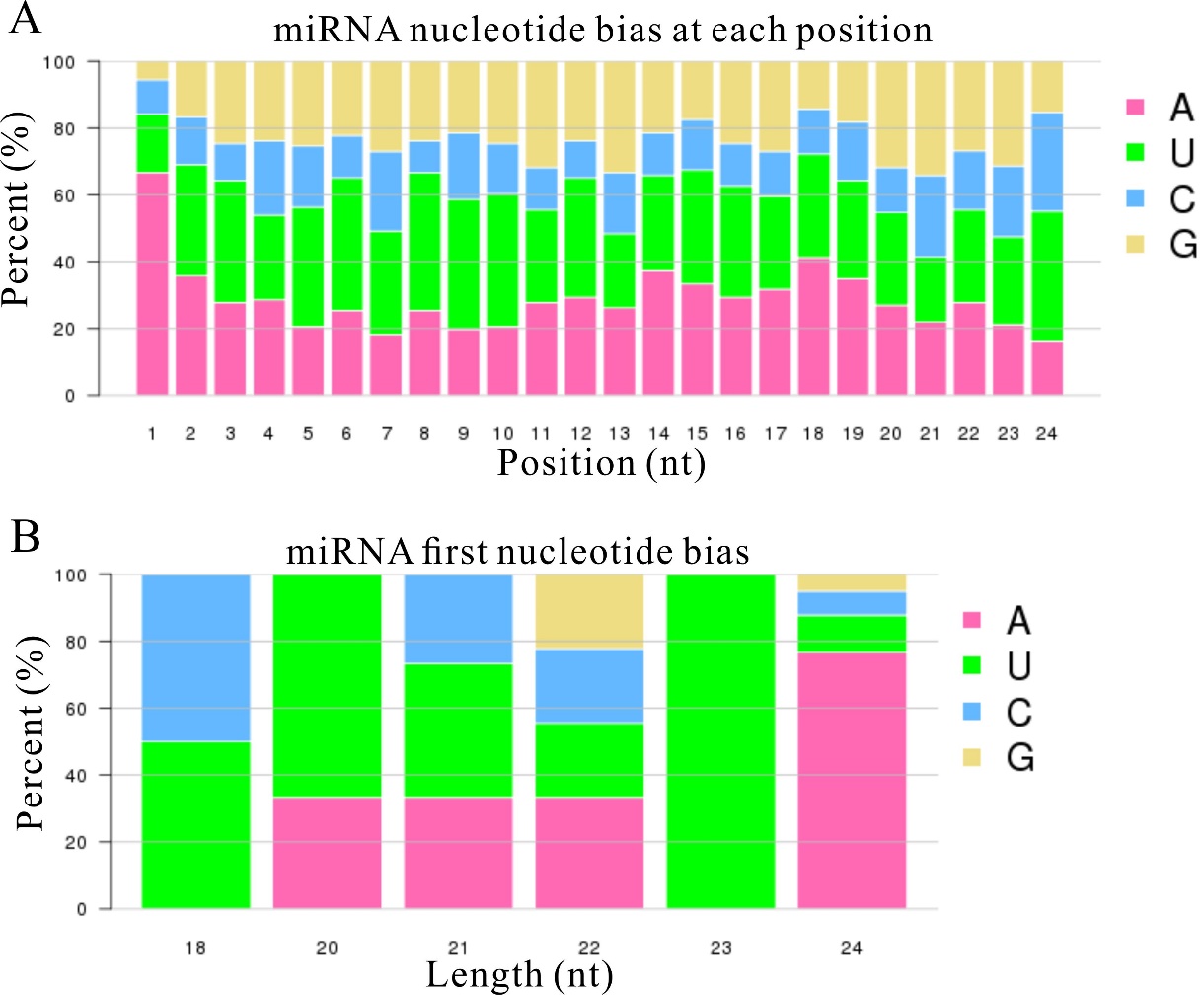


**Supplementary Figure 2.** The nucleotide bias analysis of total miRNAs from small RNA-seq. **(A)** Analysis of miRNA nucleotide bias at each position; **(B)** miRNA first nucleotide bias analysis.


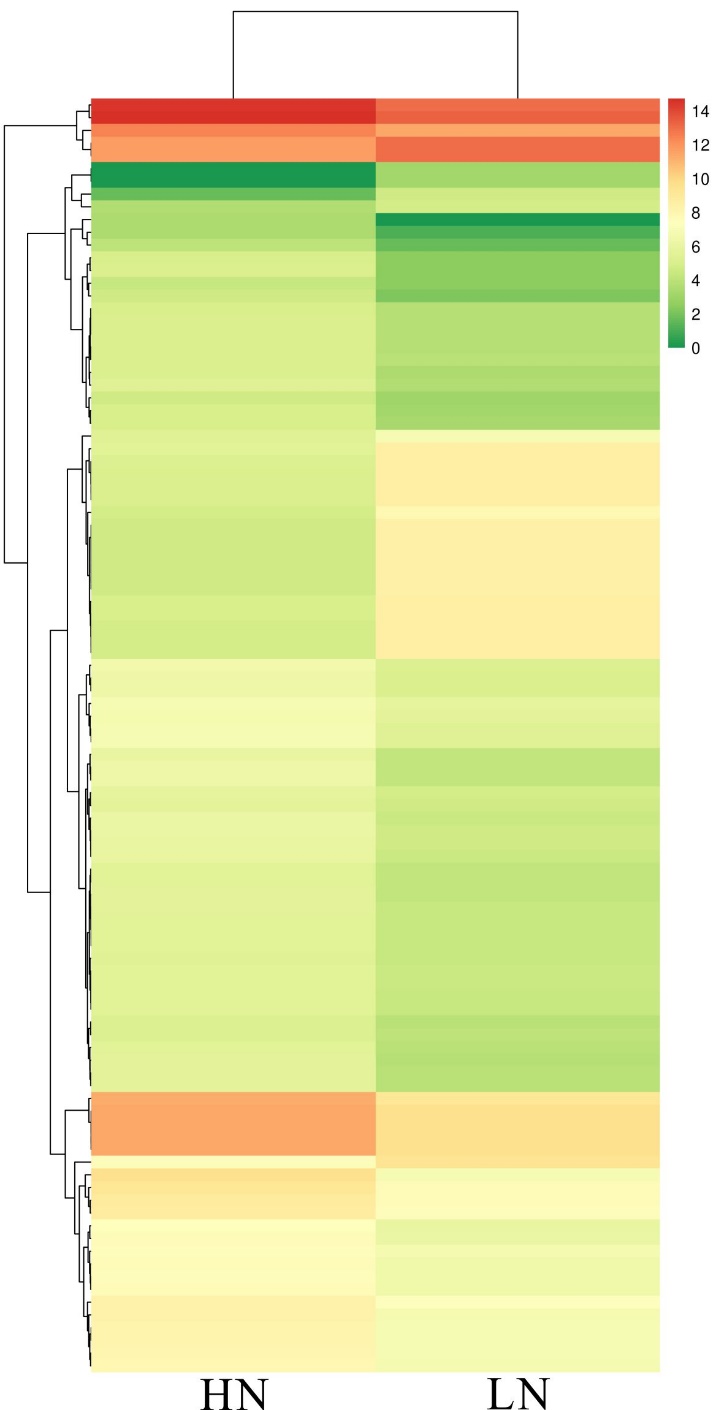


**Supplementary Figure 3.** Hierarchical cluster analysis of differentially expressed miRNAs under HN and LN conditions.


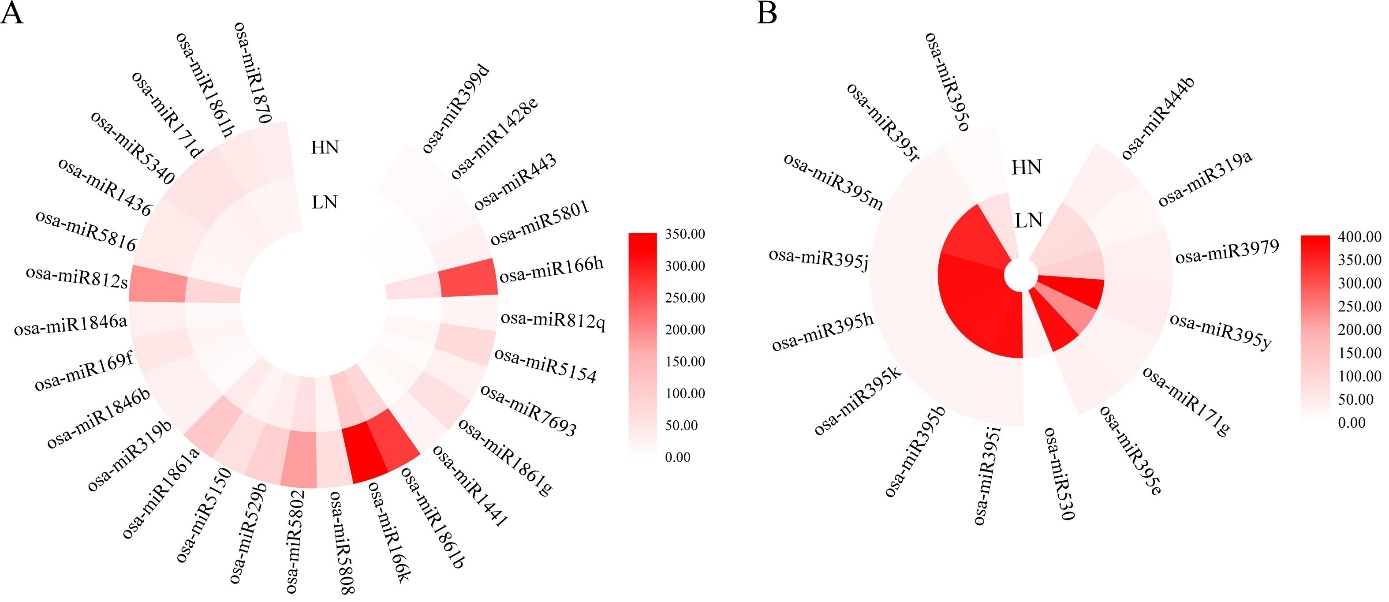


**Supplementary Figure 4.** Heatmap analysis for expression (FPKM) levels of differentially expressed miRNAs under HN and LN conditions. **(A)** Upregulated expressed miRNAs under HN condition; **(B)** Downregulated expressed miRNAs under HN condition.


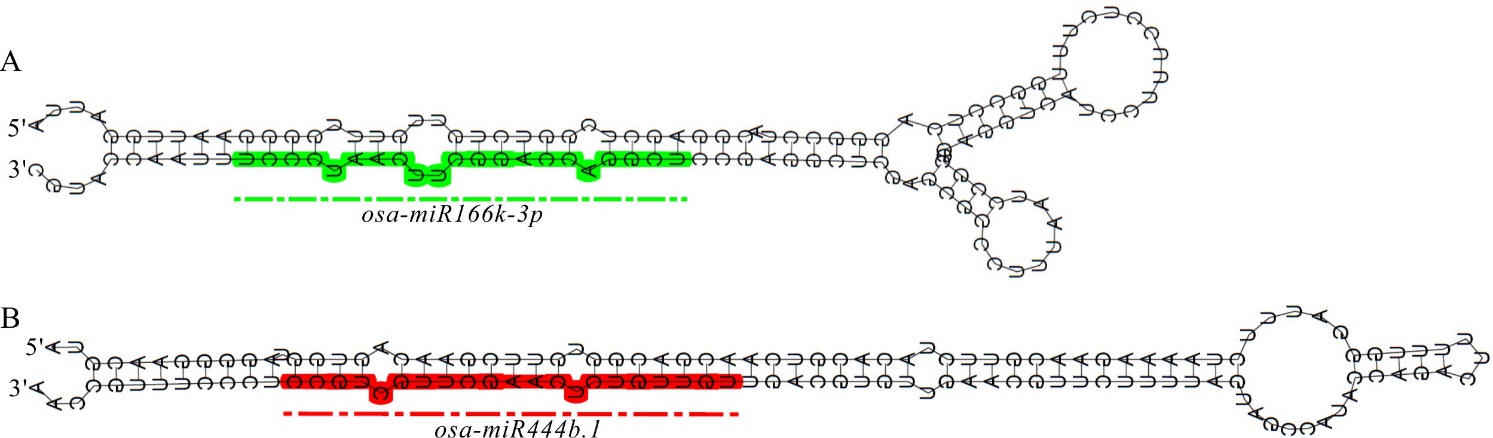


**Supplementary Figure 5.** The secondary structures prediction of *miR166k-3p* and *miR444b.1* precursors. **(A)** The secondary structure of *miR166k-3p*; **(B)** The secondary structure of *miR444b.1*.

## Supplementary Tables

**Supplementary Table 1.** Transcriptomic reads mapped to rice genome.

| Types | Total  Reads | Mapped  Reads | | Mapped  Reads(+) | Mapped  Reads(-) |
| --- | --- | --- | --- | --- | --- |
| LN | 13701210 | | 3696804 | 2425227 | 1271577 |
| HN | 17165242 | | 6068484 | 3919720 | 2148764 |

**Supplementary Table 2.** Prediction of target genes for miRNAs.

| Types | All_miRNA | miRNA_with_Target | Target_gene |
| --- | --- | --- | --- |
| Known_miRNA | 483 | 296 | 539 |
| Novel_miRNA | 128 | 32 | 1460 |
| Total | 611 | 328 | 1980 |

**Supplementary Table 3.** Prediction of target genes of *osa-miR166k-3p*.

| Target ID | Expectation | | Inhibition | Target Description |
| --- | --- | --- | --- | --- |
| LOC_Os04g49300 | | 2.0 | Cleavage | Retrotransposon protein |
| LOC_Os03g56920 | | 2.0 | Cleavage | Expressed protein |
| LOC_Os01g50230 | | 2.0 | Cleavage | Expressed protein |
| LOC_Os04g50840 | | 2.0 | Cleavage | Expressed protein |
| LOC_Os03g16320 | | 2.0 | Cleavage | Expressed protein |
| LOC_Os04g44380 | | 2.0 | Cleavage | Expressed protein |
| LOC_Os10g33960 | | 2.0 | Cleavage | Homeodomain containing protein |
| LOC_Os03g01890 | | 2.0 | Cleavage | Homeodomain containing protein |
| LOC_Os03g43930 | | 2.0 | Cleavage | Homeodomain containing protein |
| LOC_Os12g41860 | | 2.0 | Cleavage | Homeodomain containing protein |
| LOC_Os11g45899 | | 2.5 | Cleavage | Retrotransposon protein |
| LOC_Os08g36660 | | 2.5 | Cleavage | Expressed protein |
| LOC_Os01g33740 | | 2.5 | Cleavage | Retrotransposon protein |
| LOC_Os02g49670 | | 2.5 | Cleavage | Zinc knuckle family protein |
| LOC_Os03g44835 | | 2.5 | Cleavage | Expressed protein |
| LOC_Os01g08520 | | 3.0 | Cleavage | DUF581 domain containing protein |
| LOC_Os03g10810 | | 3.0 | Cleavage | HLS, putative, expressed |
| LOC_Os03g10290 | | 3.5 | Cleavage | Expressed protein |
| LOC_Os12g43900 | | 3.5 | Cleavage | Retrotransposon protein |
| LOC_Os04g49890 | | 3.5 | Cleavage | Multidrug resistance-associated protein |
| LOC_Os03g35380 | | 3.5 | Cleavage | Retrotransposon protein |
| LOC_Os03g40084 | | 3.5 | Cleavage | Expressed protein |
| LOC_Os09g35690 | | 3.5 | Cleavage | Zinc RING finger protein |
| LOC_Os11g36060 | | 3.5 | Translation | THUMP domain-containing protein |
| LOC_Os02g12730 | | 3.5 | Cleavage | Beta-galactosidase precursor |

**Supplementary Table 4.** Prediction of target genes of *osa-miR444b.1*.

| Target ID | Expectation | | Inhibition | Target Description |
| --- | --- | --- | --- | --- |
| LOC_Os02g36924 | | 0.0 | Cleavage | OsMADS27 - MADS-box family protein |
| LOC_Os04g38780 | | 0.0 | Cleavage | Transcription factor |
| LOC_Os02g49840 | | 1.0 | Cleavage | OsMADS57 - MADS-box family protein |
| LOC_Os08g33488 | | 1.0 | Cleavage | OsMADS23 - MADS-box family protein |
| LOC_Os02g17500 | | 1.5 | Cleavage | Transporter family protein |
| LOC_Os05g12770 | | 2.0 | Cleavage | NB-ARC domain containing protein |
| LOC_Os11g28070 | | 2.5 | Cleavage | Retrotransposon protein |
| LOC_Os03g47149 | | 2.5 | Cleavage | Expressed protein |
| LOC_Os04g47590 | | 2.5 | Cleavage | Niemann-Pick C1 protein precursor |
| LOC_Os05g08410 | | 3.0 | Cleavage | ATP10 protein |
| LOC_Os11g42090 | | 3.0 | Cleavage | Leucine Rich Repeat family protein |
| LOC_Os10g33840 | | 3.0 | Cleavage | Expressed protein |
| LOC_Os11g33394 | | 3.0 | Cleavage | Protein of unknown function protein |
| LOC_Os02g53260 | | 3.0 | Cleavage | Acetyltransferase, GNAT family protein |
| LOC_Os01g32849 | | 3.0 | Cleavage | Expressed protein |
| LOC_Os04g24430 | | 3.0 | Cleavage | Sucrose synthase |
| LOC_Os04g17650 | | 3.0 | Cleavage | Sucrose synthase |
| LOC_Os11g10060 | | 3.0 | Cleavage | Transcriptional corepressor SEUSS |
| LOC_Os05g40050 | | 3.0 | Cleavage | Receptor-like protein kinase 2 precursor |
| LOC_Os10g11190 | | 3.0 | Cleavage | Retrotransposon protein |
| LOC_Os10g24310 | | 3.0 | Cleavage | Retrotransposon protein |

**Supplementary Table5.** Primers used in this study.

| Primer name | Sequence (5’-3’) |
| --- | --- |
| *osa-miR166k-3p*-F | GCGTCGGACCAGGCTTCA |
| *osa-miR166k-3p*-R | AGTGCAGGGTCCGAGGTATT |
| *osa-miR395v*-F | CGCGGTGAAGTATTTGGCG |
| *osa-miR395v*-R | AGTGCAGGGTCCGAGGTATT |
| *osa-miR443*-F | CGCGCGATCACAATACAATAAA |
| *osa-miR443*-R | AGTGCAGGGTCCGAGGTATT |
| *osa-miR444b.1*-F | GCGCGTGTTGTCTCAAGCTT |
| *osa-miR444b.1*-R | AGTGCAGGGTCCGAGGTATT |
| *osa-miR530-5p*-F | CGCGTGCATTTGCACCTG |
| *osa-miR530-5p*-R | AGTGCAGGGTCCGAGGTATT |
| *osa-miR812q*-F | GCGACGTTGGGTACGAATATC |
| *osa-miR812q*-R | AGTGCAGGGTCCGAGGTATT |
| *osa-miR1441*-F | GCGACCGGATGTCGGAAA |
| *osa-miR1441*-R | AGTGCAGGGTCCGAGGTATT |
| *osa-miR1861b*-F | CGCGATCTTGAGGCAGGA |
| *osa-miR1861b*-R | AGTGCAGGGTCCGAGGTATT |
| *osa-miR5801a*-F | CGACCAAATCGTTTTCGATC |
| *osa-miR5801a*-R | AGTGCAGGGTCCGAGGTATT |
| *U6*-F | CGATTTGTGCGTGTCATCCTT |
| *U6*-R | GGGACATCCGATAAAATTGGAA |
| *HOX10*-F | ATTGATTGGGTCCAGATGCC |
| *HOX10*-R | GCCACAATACCAACCGAAT |
| *MADS27*-F | GGGGAAGATTGTGATCCGCC |
| *MADS27*-R | AGACGTGGTCCTTCTTTGTACG |
| *ACTIN1*-F | ACCATTGGTGCTGAGCGTTT |
| *ACTIN1*-R | CGCAGCTTCCATTCCTATGAA |
| *UBQ5*-F | ACCACTTCGACCGCCACTACT |
| *UBQ5*-R | ACGCCTAAGCCTGCTGGTT |
